# How Do Age and Attitudes Affect the Quality of Data Collected by Young Citizen Scientists in an Ecological Research Project?

**DOI:** 10.1002/ece3.70428

**Published:** 2024-10-10

**Authors:** Tuomas Aivelo

**Affiliations:** ^1^ Science Communication & Society, Institute of Biology University of Leiden Leiden The Netherlands; ^2^ Organismal and Evolutionary Biology Research Program University of Helsinki Helsinki Finland

**Keywords:** observations, participatory methods, school science, science education, species distribution

## Abstract

Citizen science is increasingly used to collect ecological data. Specifically, participation of school students in authentic research has been suggested as having a multitude of benefits from serving as data collection to providing science education. Nevertheless, the overall quality and quantity of data is concerning for ecologists who are using data for research. In the Helsinki Urban Rat Project, lower‐ and upper secondary school students (13–19‐year‐old) collect data on urban rat occurrence using track plates that record rat footprints. I measured the success of school‐aged citizen scientists in collecting and submitting data, and I determined the accuracy of the data they submitted by comparing their results to the results from professional researchers. Furthermore, I used additional questionnaire to relate success and accuracy to student attributes, including age, attitudes about biology as a school subject, interest in the environment and disgust sensitivity toward rats. I learned that, in contrast to results from previous studies, age was not a significant variable but rather available support from a teacher and voluntary participation with rewards were associated with higher data quality. Additionally, attitudes played a part in observer quality: higher liking of biology as a school subject was associated with lower accuracy, whereas a higher interest in the environment was associated with higher accuracy. The young citizen scientists provided broadly accurate data, although false‐positive observations were comparatively common. The results suggest that the quality and quantity of citizen‐generated data are not straightforwardly dependent on the selected target groups. Citizen science activities should be planned by careful consideration of the context as, for example, the organization of the participation strongly shapes the participatory activities.

## Graphical abstract

The young participants provided broadly good quality data in ecological citizen science project. Surprisingly, the age did not affect the data quality whereas the students who were more interested in biology as a school subject provided lower quality data than those less interested. The practicalities of the participation were important in ensuring high quality data.

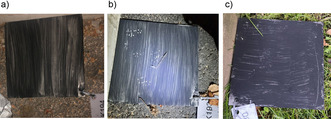

## TIIVISTELMÄ

Kansalaistiede on yhä useammin käytetty ekologisen tutkimusaineiston keruumenetelmä. Erityisesti koululaisten osallistamista aitoon tutkimukseen on suositeltu monesta syystä, lähtien aineiston laajemmasta keruusta tiedekasvatuksellisiin etuihin. Joka tapauksessa, ekologit ovat huolissaan kansalaisten keräämän aineiston laadusta ja määrästä. Vedän kansalaistiedehanketta, Helsingin kaupunkirottaprojektia, jossa yläkoululaiset ja lukiolaiset (eli 13‐19‐vuotiaat) keräävät tietoa kaupunkirottien esiintymisestä käyttämällä jälkilevyjä, joihin rottien tassunjäljet jäävät. Voin arvioida koululaisten ja lukiolaisten lähettämän aineiston tarkkuutta vertaamalla heidän arvioitaan ammattitutkijoiden arvioihin. Lisäksi käytin taustatietokyselyä, jolla pystyin tutkimaan, miten aineistonkeruun tarkkuus korreloi taustamuuttujiin, kuten ikään, kiinnostukseen biologiaan oppiaineena, kiinnostukseen ympäristöä kohtaan, ällötysherkkyyteen rottia kohtaan sekä asenteisiin rottia kohtaan. Aiemmista tuloksista poiketen ikä ei ollut merkittävä tekijä, vaan lähinnä tavat, jolla osallistuminen oli organisoitu, sillä opettajan tarjoama tuki ja osallistumisen vapaaehtoisuus lisäpalkintojen kera olivat yhteydessä tarkempiin tuloksiin. Yliedustettuja vääriä positiivisia havaintoja lukuun ottamatta nuorten kansalaistieteilijöiden keräämä aineisto oli luotettavaa. Lisäksi asenteet vaikuttivat tuloksiin, sillä biologiasta pitäminen oppiaineena johti heikompaan tarkkuuteen, kun taas positiivinen ympäristöasenne johti parempaan tarkkuuteen Tulokseni osoittavat, että kansalaislähtöisen aineiston määrä ja laatu ei suoraan riipu valituista kohderyhmistä. Tutkimuksen viitekehyksen huomioiminen on olennaista, koska esimerkiksi opettajien ratkaisut osallistumisesta muovaavat vahvasti osallistumisen tapoja.

## 1 Introduction

Citizen science is increasingly used to generate data and knowledge of the natural world (Bonney et al. 2009). Indeed, ecology has been using citizen‐generated data and knowledge for a long time (Dickinson et al. 2012; Pocock et al. 2017): Much of the species occurrence data is collected by citizen observations, ranging from experienced amateurs, such as bird enthusiasts, to less experienced citizens who provide photographs on social media (McKinley et al. 2017; Miller‐Rushing, Primack, and Bonney 2012). The quality of this citizen science data and knowledge and usability for further analyses has been addressed in many studies (Dickinson, Zuckerberg, and Bonter 2010; Isaac et al. 2014; Johnston, Matechou, and Dennis 2022; Kelling et al. 2015; Lukyanenko, Parsons, and Wiersma 2016).

Citizen science projects range from highly technical to simpler projects: Some projects require specialist knowledge and long‐term commitment whereas other can be easily participated in for a short period without specific training or preparation (Pocock et al. 2017): For example, iNaturalist observations could require only uploading data on individual observation, whereas bird ringing requires specific training and identification skills that take time to master. Consequently, the quality of the citizen‐collected data varies substantially depending on the project (Aceves‐Bueno et al. 2017; Kosmala et al. 2016) and the protocol chosen by a researcher is often a limiting factor in relation to the quality and quantity of the collected data (Dickinson, Zuckerberg, and Bonter 2010). Citizen science data have been validated with internal and external datasets (Matutini et al. 2021) or compared citizen participants' assessments with professional assessments (Di Cecco et al. 2021; Gorleri et al. 2023; Kelling et al. 2015). These studies have shown that understanding citizen scientists' behavior is crucial in understanding biases and error rates arising from the data collection processes. The quality of the citizen scientist collected data can be improved prior to the data collection by targeting and choosing the participants or through training of the participants, while after data collection, the known biases can be considered during the analysis (Johnston, Matechou, and Dennis 2022). Indeed, there are different guidelines for researchers on how to choose and approach the target group of participating citizens to maximize data quality or optimize it in relation to data quantity. The characteristics of the participants are considered through *observer quality*, that is, how reliable data is produced by the participating citizen (Dickinson, Zuckerberg, and Bonter 2010; Horns, Adler, and Şekercioğlu 2018; Welvaert and Caley 2016). Thus far, observer quality has been only considered through technical skills either directly (Genet and Sargent 2003; Sauer, Peterjohn, and Link 1994) or through the proxy of age apparently for the ease of data collection (Delaney et al. 2008). While the effects of participation in the citizen science projects on participants' attitudes have been previously studied (Bonney et al. 2016; Brossard, Lewenstein, and Bonney 2005; Kelemen‐Finan, Scheuch, and Winter 2018), to the best of my knowledge, only one study has previously examined how participants' attitudes affect the collected data quality: Crall et al. (2011) compared citizen scientists versus professionals in a simulated setting identifying invasive plant species, mapping their distributions, and estimating their abundance while measuring scientific literacy and attitudes. Indeed, it would be expected that not only skill but also motivations and interest would shape the outcomes of participation, but Crall et al. (2011) found that these were poor predictors of data collection quality and could not be used as eligibility criteria.

### 1.1 Ecological Citizen Science and Attitudes

An attitude is a disposition toward a particular concrete or abstract object, person, thing, or event with favor or disfavor (Eagly and Chaiken 1993). The attitudes can be single or multiple constructs: For example, attitudes toward science learning consists of numerous subconstructs. The attitudes are expected to lead to motivation and behaviors. Previous research has shown that in animal‐related citizen science projects, the interest or affection toward the target species is one of the factors that drives participation and motivation to collect citizen science data (Land‐Zandstra, Agnello, and Selman Gültekin 2021). For example, “charismatic megafauna” are conjured in conservation contexts to create affection between humans and wildlife (Monsarrat and Kerley 2018). In contrast, some biodiversity is considered disagreeable and even disgusting, such as the focal species in this project, rats (Bird Rose and van Dooren 2011; Davey 1994; Prokop and Tunnicliffe 2008). Disgust sensitivity is a rather versatile affect, which can also drive interest and transform into more positive effects and even drive learning and interest (Davey 2011; Kolnai 2004; Prokop and Fančovičová 2017; Randler, Hummel, and Wüst‐Ackermann 2013); thus, this suggests that while participants might have negative attitudes toward species perceived as disgusting, they can nevertheless still stimulate participants' interest.

The school‐situated citizen science projects have been widely studied because they are usually the most common context in which young citizens participate in citizen science. In addition to data quality, there are many studies on the learning outcomes as the incorporation of citizen science into formal education requires considering the school curriculum (Cronje et al. 2011; Dickerson‐Lange et al. 2016; Hadjichambi et al. 2023; Hiller and Kitsantas 2014; Kelemen‐Finan, Scheuch, and Winter 2018; Shah and Martinez 2016; Trumbull et al. 2000). Nevertheless, the students' liking of school subjects has not been studied in relation to their participation in citizen science during these subject classes.

### 1.2 Aims and Research Questions

This study is situated in the Helsinki Urban Rat Project (HURP—https://www.helsinki.fi/en/projects/urban‐rats), which provides a unique setting to understand the interplay between young citizens' attitudes, age, and the quality of generated data. HURP studies rat population dynamics and models spatiotemporal variation in rat occurrence around the city of Helsinki in Southern Finland. The study organism is the brown rat (*Rattus norvegicus*), about which humans usually have strong negative attitudes (Bjerke and Østdahl 2004; Kellert 1985). I have organized a data collection with young citizens collecting rat presence–absence data with track plates (Hacker et al. 2016); all ecological data is double‐checked by professional scientists.

I explored the data quality and operationalize observer quality with two different approaches: I measured the success rate of the participants and compared the young citizens' assessments of the track plates and how well it matched with the expert assessment in both presence/absence of the tracks and the number of tracks. Then I matched this data into participant questionnaire data and explored how the participants' attitudes, gender, received support from teachers and age correlated with the success and accuracy of data collection.

I hypothesize that older students would have higher accuracy and success rate compared with younger students as they would have better general science research skills. Additionally, I would expect available support from teachers, such as dedicated time to discuss tracks on plates, to increase both accuracy and success of data collection, whereas voluntary participation would mean that only the most motivated students would participate; thus, the accuracy would be increased. Regarding the attitudes, I would expect that the accuracy and success rate would be higher for participants who are more interested in studying biology, have more proenvironmental attitudes and who are more positive about rats as all these attitudes potentially lead to more careful and considerate data collection. Similarly, I would expect that lower disgust toward rats would increase success rate and accuracy as it would suggest more affection toward rats. I would expect that similar processes would operate on both abilities to collect data at all and the identification or count errors.

## 2 Materials and Methods

The project has been running since April 2018, and the data used here has been collected until the end of 2021. I recruited lower‐ and upper secondary school biology teachers through a Facebook group by selecting all 33 teachers from 20 schools who had responded to my post. Teachers were not specifically trained prior to the start of the project. The project participation for individual schools began with the researcher's visit, where I brought the necessary materials and gave a 1‐h classroom lecture for the teacher and students. The lecture included a general introduction to urban ecology, background on rats as species including their movement habits in the urban environment, aims of the study and guidance on how to use the track plates and send collected data. This information was also available at the project website. This project fitted Finnish curricula in two counts: Urban or anthropogenic habitats are listed as concepts and inquiry‐based or experimental coursework and scientific inquiry skills are emphasized in both lower‐ and upper secondary schools. Teachers were then free to organize their group's participation as suited them best, and no specific follow‐up was provided after the lecture. At the end of each term, I sent an email to teachers asking about the data collection, specifying any submitted data which was incomplete and asked teachers to circulate the link for the project questionnaire to their students.

The participating lower secondary school students were 13‐ to 16‐year‐olds, where the mode was 15‐year‐olds, whereas the upper secondary school students were 16‐ to 19‐year‐olds with 17 as the mode. Teachers usually participated multiple times (1–7 times) with different student groups and did not have time to prepare much for the project; thus, the first time they participated was usually not well planned. I visited lower secondary schools 23 times and upper secondary schools 56 times. In this article, I use students and young citizen scientists interchangeably to acknowledge the overlapping roles of students who are participating not only as a part of school assignments but also as citizen scientists who are creating scientific knowledge and understanding themselves that this project was also different from an ordinary classroom assignment.

Based on the discussions with teachers, the broader conversations related to the project in following lessons varied vastly. Due to my focus on the data quality, I only collected data on the process up until the data collection. As the schools had different methods of organizing their participation and data collection, I surveyed teachers to determine whether students (1) received support in analyzing and counting track plate markings as this would be expected to affect the data quality and (2) whether participation was obligatory for students and whether it affected the grading of the coursework as this would affect which students participate. I categorized teachers' answers in both variables to three classes: The participation could be (i) mandatory, (ii) voluntary without extra credit or (iii) voluntary with students receiving extra credit, while teacher support was classified as (i) teachers provided no additional support, that is, the students had to independently interpret the track plates, (ii) teachers provided help in interpreting track plate tracks during the lessons, if students asked for, or (iii) students identified and counted rat tracks together in the classroom in an activity led by a teacher.

### 2.1 Track Plates and the Success and Accuracy of Citizen Scientists

**FIGURE 1:**
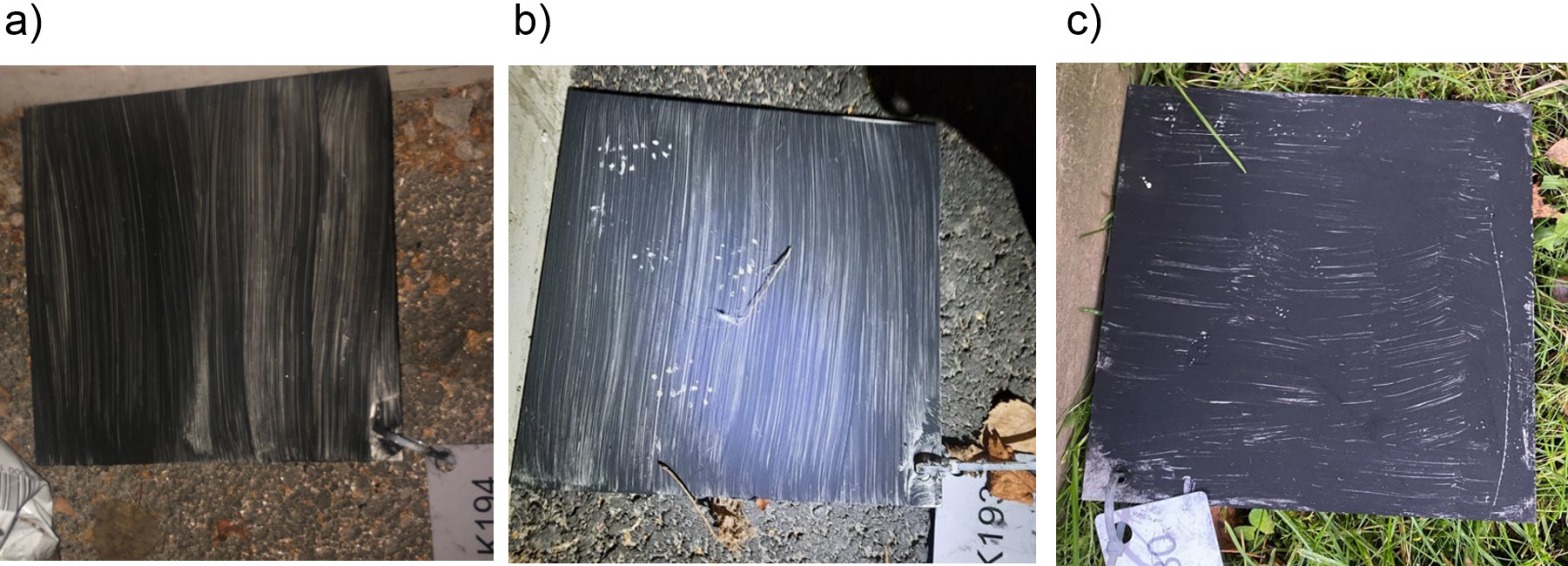
Examples of track plates (a) without any tracks, (b) with rat tracks, and (c) with tracks of smaller rodent, that is, mice or vole. Photographs are taken by the participants of the project and the project has been given the publishing rights for all submitted photographs.

Rat presence or absence was assessed with track plates (Figure 1). The minimum requirements for participation were that there were four plates within one study area, and they were photographed daily for 4 days after setting the plates. When sending the data, the students counted rat tracks on the plates by dividing the plate to a 5 × 5 grid and observing how many grid squares had rat tracks; thus, they would submit a score between 0 and 25 for each plate on each of the four observation days. The data were submitted to the database through the EpiCollect5 mobile application (Aanensen et al. 2009). In general, the students were free to decide where they set the plates and whether they worked in groups or as individuals. I did not collect data on whether the students worked in groups or alone, but post hoc assessed the effects based on proxy data (Supporting Information, Figure S1).

The total number of data collection was 3006 plates, of which 641 were collected by lower‐ and 2365 by upper secondary school students leading to an average 24 and 41 plates per visit, respectively (Table S1). After the student participation, HURP researchers checked the data integrity. The submissions that did not include photographs of the plates or had missing values on location or date were deleted and considered as unsuccessful data collection. The researchers identified any tracks on the plate and noted the number of grids with rat tracks. Thus, for each plate there was a number of observed (by a young citizen scientist) and confirmed (by an experienced researcher) rat tracks. The count accuracy of young citizen scientists was measured as the congruence between the observed and confirmed tracks. The accuracy was measured as the sum of differences between observed and confirmed counts divided by the number of the plates. Thus, 0 would mean full concurrence (i.e., highest accuracy) whereas the maximum possible value (i.e., lowest accuracy) would be 100 (which corresponds to 4 days times 25 per day).

I assessed the presence–absence accuracy of the data collection by assessing the number of true and false positives and true and false negatives. True positives are plates that had confirmed rat tracks and citizen scientists had identified these as rat tracks. False positives are plates that did not have rat tracks, but on which citizen scientists thought there were rat tracks. True negatives are plates which the citizen scientists correctly reported no rat tracks and false negative are plates on which there were rat tracks, though the citizen scientists reported no tracks. This allowed me to calculate the presence/absence accuracy (true negatives and positives divided by all observations), precision (true positives divided by true and false positives), sensitivity (true positives divided by true positives and false negatives), and specificity (true negatives divided by true negatives and false positives) of presence or absence of rats in study sites.

### 2.2 Questionnaire for Participants

Approximately 2500 students took part in the data collection, and we had a total of 772 responses to the questionnaire; thus, we had a response rate of approximately 31%. The postparticipation questionnaire asked students the identification codes of track plates that had been used by the participants when submitting data; thus, the data on the observed and confirmed number of tracks in the plates were manually added to this data. If there was a clear mention of the plates used, but they were not found in the database (i.e., they were removed during quality control) or they belonged to deleted entries, I noted these as having zero plates; therefore, they amounted to unsuccessful data collection.

**FIGURE 2:**
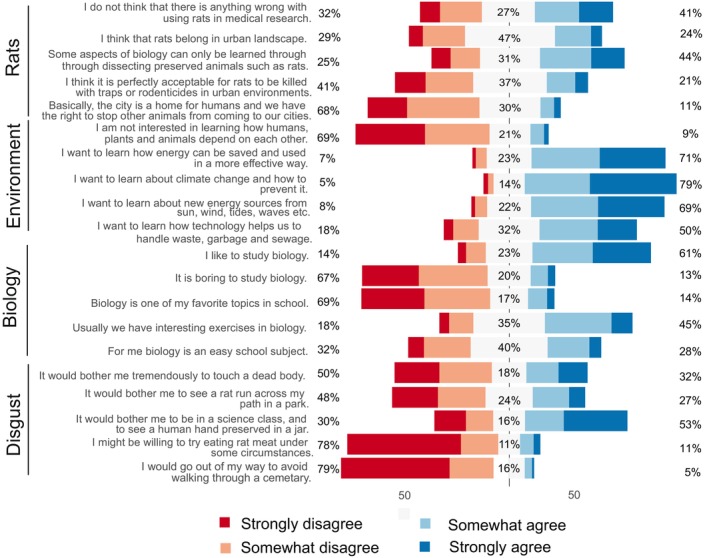
Distribution of the responses for each item grouped by scales (left side). The value on the left side indicated proportions of respondents who strongly or somewhat disagree, those in the middle did not disagree or agree. Respondents on the right strongly or somewhat agree. The item‐related statistics are shown in Tables S2 and S3.

To understand student attitudes for this study, four different instruments with five items per scale were combined in a questionnaire (Figure 2). I expected these previously developed and tested instruments to explain how students approached the task and how reliable the data were that they were able to collect. As the students participated in the citizen science project during compulsory biology lessons, I selected the liking of biology as a school subject as one of the focal attitudes as it could modify the students' motivation in participating and collecting data. *Liking of biology as school subject* is based on the modified version of Fennema‐Sherman Mathematics Attitude Scales (Fennema and Sherman 1976; Metsämuuronen 2012) used in Finnish national assessments. Furthermore, the spatial context of the participation was the students' own near environment, such as school or their own homes, and we know from previous research that this is considered as an important aspect of student motivation (Aivelo and Huovelin 2020); thus, interest in learning about environmental issues should illuminate how students approach using scientific equipment and their own knowledge about their living area. *Interest in Environmental Issues* was based on the international ROSE (The Relevance of Science Education) questionnaire (Schreiner and Sjøberg 2004) in which these items form a singular factor (Uitto et al. 2011). *Attitude toward Rats* was a modified scale based on an Animal Attitude Scale (Herzog, Grayson, and McCord 2015). *Disgust sensitivity* is based on the items from the revised Disgust Scale (Haidt, McCauley, and Rozin 1994; Olatunji et al. 2007). These items form subscales of Core disgust and Animal Reminder. The scales were piloted by the first two participating groups, and they were deemed to perform satisfactorily, that is, items were not multifactorial and had a satisfactory explanatory values and respondents had consistent answers (see section 2.3).

### 2.3 Statistical Analysis

To calculate individual factors loadings for each of the expected attitude scales, I used item response theory (IRT). IRT allows for multidimensional latent trait modeling and considers each item individually; thus, it allows for items in the same scale to have different response curves for latent traits. I used a generalized partial credit model (GPCM; Muraki 1992) with Metropolis‐Hastings Robbins‐Monro (MH‐RM; Cai 2010a, 2010b) algorithm implemented in a mirt package (Chalmers 2012) in R (R Core Team 2013). MH‐RM combines the flexibility of a Bayesian approach to computationally lighter maximum likelihood estimation (Cai 2010b). GPCM is a constrained graded model that is adjusted by only a single “difficulty” parameter. I assessed the usability of the factors by first doing an exploratory model, where all items were allowed to freely load on four different factors. After running the exploratory model, I removed those items that loaded clearly on multiple factors and had low explanatory value (as measured by *h* ^2^). In exploratory IRT modeling, I recovered the expected factors, though not all factors performed optimally: items 9 and 20 had low factor communality values; therefore, they were unreliable and dropped (Table S2). 17 respondents did not fit the model due to inconsistent answers to the items within the scales; they were also removed from the following steps in the analysis.

Next, I performed a confirmatory modeling, in which I forced the items to load on predetermined factors. I assessed the model fit, item fit, and personal fit as with the exploratory model. I removed the outlying respondents and redid the confirmatory analysis. The confirmatory model shows an adequate fit (RMSEA = 0.062 (0.054–0.071), TLI = 0.88, CFI = 0.91; Table S2). Using the factor scores for each respondent, I further modeled accuracy through the covariance of four aforementioned scales.

To model the success and accuracy of collecting data, I built generalized linear mixed models that can consider both random effects and model different response variable distributions. The school was used as a random factor while age, gender, teacher support, voluntariness, and factor scores for liking of biology, attitude toward rats, interest toward environmental issues and disgust were fixed factors. The responding variable was either the success of collecting data or the accuracy. The former included all the respondents for the questionnaire and the value was either success (1) or failure (0) using a binomial model. The latter was a numerical value between 0 and 25 and the dataset included all respondents who were successful in collecting data.

The full dataset is deposited in FigShare (10.6084/m9.figshare.19583206), whereas the code for the analysis is deposited in GitHub: https://github.com/aivelo/citsci.

### 2.4 Ethical Considerations

Participation in the data collection for the citizen science project was part of the regular schoolwork; therefore, the students had to participate in it with the exception of four schools where it was a voluntary part of the coursework (and in two of these, the students were rewarded for participating, e.g., with extra points toward the course grade). Furthermore, in three schools, the data collection included scientific report writing or other additional assignments that could affect the students' grades. The questionnaire instructions mentioned that responding to the questionnaire was voluntary, participation could be ended at any time, no data would be given to their teachers and response or lack of response to the questionnaire would not affect their grades. The first question of the questionnaire was aimed to record the informed consent of the students and 24 students did not give their consent at this stage, suggesting the students did have free choice in the questionnaire even if they had done it in the context of regular schoolwork.

Based on Finnish ethical review guidelines, there was no institutional prerequisite for an ethical review at University of Helsinki. The research permits were granted by the City of Helsinki on April 5, 2018, and September 22, 2020, respectively, and the permits from individual private schools on May 3, September 7, and October 1, 2018. All participants were informed about the aim of the study and how the materials would be collected, stored, and handled anonymously. The parents were informed beforehand in the case of students being under 15 years of age to have the possibility to opt out, while the older students could give their own consent to participate. All personal data were collected anonymously by the HURP during the study and the linking of the track plates, and the questionnaire responses was done through track plate codes. In cases where teachers used track plates as a part of their assessment of the coursework, no information about the names was handled by HURP at any point. There was a potential risk, albeit very small, due to the amount of data, if the students collected the data from their own home yard, that the data collection could be linked to individual data; therefore, students were advised not to do this, if they were concerned.

## 3 Results

### 3.1 The Participants and the Success of Collecting Data

There was a total of 772 responses to the questionnaire, of which 645 had consented to take part, completed data so that we could connect the responses to the track plate data sent through EpiCollect app and there were at least 10 responses per school. These respondents represented 1487 plates (50% of the total plates). There was a total of 12 schools (with a range of 10–224 respondents per school). The mean age of respondents was 16.56 (standard deviation ±1.14); 53% were females, 43% males, 1% identified as others, and 3% did not want to identify their gender. The respondents were generally positive toward learning biology (e.g., only 14% disagreed with the statement “I like to study biology;” Figure 2) and were very positive toward learning about environmental issues (e.g., only 9% agreed with “I am not interested in learning how humans, animals and plants depend on each other.”) In contrast, the attitude toward rats was much more uniformly distributed; for example, for the statement “I think it is perfectly acceptable for rats to be killed with traps or rodenticides,” 41% of the students disagreed and 21% agreed.

I was able to use 72% of the respondents (463/645) for the assessment of data quality; that meant that the plates were in an acceptable place, there was data for at least one night, the photos were clear enough to assess, and I was able to link their questionnaire response to their track plate data.

### 3.2 The Accuracy of Participant Collected Data

In the presence/absence data collected by the participants, the most common class was true negative occurrences, that is, those classified as not having rat tracks by both citizen scientists and ecologists, with 216 related responses (47%), while true positives, that is, classified by having rat tracks by both, were the second most common class, with 137 instances (30%). In contrast, false positives were much more common than false negatives (78 (17%) vs. 26 (6%)). The significant variables affecting true and false positive and negatives are outlined in Table S4a–d. False positives relate to the tracks left by other animals, than rats, such as mice, squirrels, or hedgehogs which were interpreted to be rats, while false negatives were plates with rat tracks that were either near the edges of the plate or otherwise difficult to note and in a minority with the cases of other animal tracks being on the plates, suggesting that students might have not noticed rat tracks among all the other tracks.

The presence–absence accuracy, meaning the proportion of concordant track analysis was 77%. Precision, that is, the proportion of correctly identified presences of all presences, was 62%. Sensitivity, that is, the probability of identifying presence correctly was 82%. Finally, specificity, that is, the probability of identifying absence correctly was 73%. These values were calculated as the professional ecologists' assessments assumed to be the “true” value; thus, they are the concordance between young citizen scientists and professional researchers.

When looking at the difference between lower‐ and upper secondary schools, I found that lower secondary schools had substantially lower values (presence–absence accuracy in upper secondary school 79% versus 62% in lower, precision 64% vs. 59%, sensitivity 89% vs. 48%, but specificity was 73% for both). Notably, the proportion of success in collecting data differed only slightly between school levels (75% in lower secondary school and 72% in upper secondary school). Similarly, the lower secondary schools had less accurate data as they had a higher discrepancy between student‐observed and researcher‐confirmed counts of rat tracks (comparing only plates with rat tracks: mean 3.59 ± SD 10.4, vs. 1.78 ± 6.87, *χ* ^2^_130_ = 192.3, *p* < 0.001).

### 3.3 The Factors Correlating With Count Accuracy or Overall Success

**FIGURE 3:**
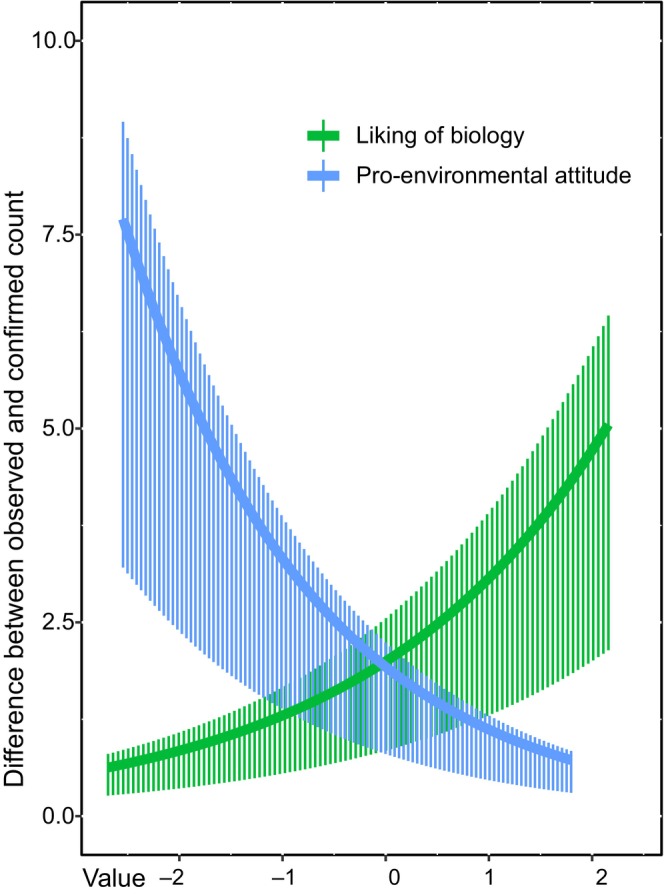
Effect sizes of the statistically significant attitude variables affecting accuracy. The x‐axis shows attitude values for liking biology and proenvironmental attitude. In the y‐axis, the higher the difference between observed (young citizen) and confirmed (expert) count were, the less accurate data. Attitudes toward biology as a school subject and environmental issues were more concentrated on positive values.

The mixed‐effects modeling showed that on the proxies for participant skill, respondent age was not a significant variable but rather the organization of participation was (i.e., help when needed, *p* = 0.03, and voluntary participation with rewards, *p* = 0.01; Table 1; both increased accuracy). On attitudes, more positive attitude toward learning about the environment was negatively correlated with the number of errors, thus meaning better count accuracy (*p* = 0.04; Table 1; Figure 3; though when considering only plates with research‐confirmed tracks, no attitude was significant, Table S5). In contrast, attitude toward biology as a school subject was significantly positively correlated, meaning that a more positive attitude toward biology led to more errors and, thus, lower quality data (*p* = 0.05, Table 1; Figure 3). When considering the overall success of data collection, the significant variables were voluntary participation with rewards (*p* = 0.02) and the track plate checking together (*p* < 0.01), both of which decreased the success rate (Table S6).

**TABLE 1:** Variables affecting the count accuracy.

	Variable		Estimate	Std. error	*z* value	*p*
	(Intercept)		1.19	0.57	2.10	0.04
	Gender	Male	0.14	0.31	0.48	0.64
		Not specified	−3.67	1.90	−1.94	0.05
		Other	−0.27	6.98	0.00	1.00
Proxies for skill	Age		0.08	0.20	0.14	0.11
	**Support**	**When needed**	**−0.73**	**0.35**	**−2.11**	**0.03**
		Done together	−1.11	0.90	−1.23	0.22
	**Choice**	**Voluntary with a reward**	**−1.24**	**0.50**	**−2.48**	**0.01**
Attitudes and disgust sensitivity	Rat		0.01	0.24	0.07	0.94
	Disgust		−0.07	0.21	−0.32	0.75
	**Biology**		**0.39**	**0.20**	**2.00**	**0.05**
	**Environment**		**−0.45**	**0.22**	**−2.04**	**0.04**

*Note:* Students who received help when needed and those whose participation was voluntary but rewarded were more accurate. Additionally, those with higher liking of biology as a school subject had lower accuracy, whereas those that had more interest in learning about environmental issues had higher accuracy. The model was a generalized mixed model where school was used as a random factor and the baseline is female who did not receive extra help with mandatory participation. The significant variables are marked in bold font.

## 4 Discussion

I approached observer quality in citizen science through technical skills (by using age as a proxy), the organization of participation and participants' attitudes. My study shows that age is a nonfactor when assessing the observer quality of 13–19‐year‐olds participating in urban ecology citizen science project. Interestingly, the attitudes of the participants were statistically significant factors: Proenvironmental attitude and interest in biology as a school subject affected the accuracy. The effect of attitudes has not previously been found to affect results in citizen science projects (Crall et al. 2011; Pagès, Fischer, and van der Wal 2017).

My results show that young citizen scientists were accurate at identifying rat tracks on the track plates; however, in contrast, they were likely to produce false positives when there were no tracks. In general, this cannot be seen as a surprise, as the project had the explicit aim of assessing rat occurrence; thus, participants likely perceived rat tracks as an implicit goal of the study (Kervinen et al. 2024). This finding is also in line with previous research where volunteers were shown to provide false positives of rare animals in camera‐trap material collected in Serengeti National Park (Swanson et al. 2016). Thus, if the use of time needs to be considered in double‐checking the citizen‐collected data, the focus in positive samples is warranted.

There was no effect of age on count accuracy or overall success. The general expectation in citizen science projects involving young citizens suggests that older participants are more skilled; thus, they are expected to provide more reliable data (Delaney et al. 2008; Dickinson, Zuckerberg, and Bonter 2010). Clearly, this effect is not universal, if the protocol is easy enough. Here, the research protocol was comparatively easy as no specialist skills were needed to participate. Thus, it would be expected in the research protocols that require more abstract thinking or technically more complex tasks, the effect of age would be noticeable. It should be noted, though, that the older students did collect more data per student than the younger students.

The organization (i.e., available help and voluntariness) of the school students' participation was a significant factor in explaining differences in count accuracy and success rate. The availability of help increased the participants' accuracy, which was not surprising, as usually the teachers had participated in the project previously; thus, they had expertise in identifying rat tracks. Similarly, voluntary participation with rewards enhanced the accuracy compared to mandatory participation. This might be more through the selection bias than actual improvement of research skills: those more motivated to achieve high grades are also more likely to participate and to provide quality data. In general, motivation to do “extra work” outside of the school hours might be lower without any rewards and lead to less effort in obtaining accurate data. Indeed, the total number of plates completed in upper secondary schools when participation was totally voluntary was minimal.

Attitudes were also significant variables. Surprisingly, in this study, those more interested in studying biology erred more often than those with less positive attitude toward biology as a school subject and they were more likely to falsely detect rat tracks. This is surprising as a positive attitude toward learning biology would be expected to mean that student would try to do more careful work and follow the given guidelines more closely (San Llorente Capdevila et al. 2020). The correlation raises the question whether the students perceive the goal of the study to be finding rats and then those students who are the most interested in the school subject are more inclined to try to fill this perceived expectation, that is, excel in the school subject. Then again, when lower secondary students were specifically asked whether they felt that the research had failed when they found no rat tracks, the students were able to differentiate between having a result of no rat tracks and not getting valid results at all (Kervinen and Aivelo 2023). Broadly, this result also suggests that enthusiasm in participants cannot be straight forwardly considered as a good issue in relation to the data quality.

Interest in environmental attitudes correlated positively with accuracy whereas disgust or animal attitudes did not correlate at all. It is not clear why a proenvironmental attitude was linked with greater accuracy. As students were prompted to think about rat presence and absence through the lens of their own near environment and to use their local knowledge, this could show a connection between learning about the environment in *general* and learning about rat presence in the near environment *specifically*. While it is difficult to assess whether studying rats was inherently more or less motivating for the students (Prokop and Fančovičová 2017; Randler, Hummel, and Wüst‐Ackermann 2013), at least their attitudes toward rats or disgust did not seem to play a part in the quality of their eventual data gathering.

### 4.1 Limitations

The questionnaire was submitted after the fieldwork had been completed. The underlying assumption here is that there is no substantial change in student's attitudes during participation. In our previous work, the student interviews have shown that the students perceive that they have more positive attitudes toward rats after the research (or at least they have reflected on their relationship with rats) (Aivelo and Huovelin 2020). Nevertheless, it is unlikely that the attitudes that are measured here change substantially during the participation (Ajzen 2001; Haidt, McCauley, and Rozin 1994; Metsämuuronen and Tuohilampi 2014), specifically as the participation in the research was not a planned intervention on student's attitudes. The participation was a rather long process which ended with the track identification and counting. The questionnaires were administered right after this data collection. Thus, I would argue that the questionnaires were given at a time point nearest to the step which resulted in the data quality that I have studied here.

I cannot assess how representative my sample is in relation to all the students in Helsinki (Table S1). While the sample contains schools with very different backgrounds, including language, location, student background, the actual respondents are probably biased toward the more motivated students as participation in the questionnaire was voluntary. Indeed, as the students generally like biology as a school subject and have proenvironmental attitudes, the variation in these attitudes is rather small.

There is a broad variety of ecological citizen science projects; this raises a question on how the results from my study can be generalized in relation to other citizen science projects. The research protocol was suitable for the students as there was a good success rate and there were no clear age‐associated effects on how successful they were in data collection. Thus, I would expect that the results of this study could be generalized to other citizen science projects in a school context.

It is beyond the scope of this article whether the data collected by the young citizens is useful for the ecological modeling of the rat occurrence as this work is still under way. HURP has two other stakeholder‐generated datasets on urban rat occurrences: Trap data from an extermination company and direct observations from trash management personnel. These can be later used to validate the young citizen‐generated dataset.

### 4.2 Practical Considerations for Citizen Science Studies in Schools

I have considered here only one aspect of the citizen science project data collection: The quality of the data provided by citizen scientists. In citizen science projects, citizens can also provide data that would be inaccessible to researchers otherwise. For example, in this project, students studied their own near environments; thus, they provided local knowledge on which they were the best experts. Indeed, successful data collection further requires the imagining of rats' experiential worlds, and I would argue that many aspects of the data collection are more than just crowdsourcing: The selection of study sites and implementation of data collection already require quite complex science skills. Thus, data quality should not be the only consideration when thinking about usability of the data but also the already existing knowledge of the participants. In ecological citizen science, this relates self‐evidently to the participants' local knowledge but it can also mean that participants' everyday experiences open new analytical potentials (Kervinen and Aivelo 2023). Indeed, any trade‐offs within the project protocol (i.e., working in groups, collecting data within walking distance from school or during off‐hours near students' own homes) need to be considered not only from data quantity or quality point of view. Results here show that the organization of the participation was an important factor, but it is not trivial to identify these differences as they are highly context‐dependent. For example, in this study, these variables were formed after student participation after repeated discussions with their teachers. I suggest that open‐ended discussions on participation with participants or in this case their teachers are vital to identifying these important aspects.

Furthermore, it is important to remember that the ability of the participants to provide data is only one of the considerations taken in the design of citizen science studies. Important aspects include science education, democratization of science practices and knowledge creation, appreciation of Indigenous or local knowledge, attitude and behavior change, empowerment, and actual change in participants' environment (Ballard, Dixon, and Harris 2017; Christine and Thinyane 2021; Rautio et al. 2022; Tengö et al. 2021). These goals could and very probably will be in conflict with scientists' interest in collecting data. Sometimes these choices come with trade‐offs: for example, in our study, the students at the lower secondary school came generally from the vicinity of the school whereas upper secondary school students come from the entire city and neighboring cities. Thus, targeting lower secondary schools allowed for more targeted areas of data collection.

School context strongly drives participation as everything needs to occur within the curriculum and school year schedule (Atias et al. 2023; Ballard 2023). Our project was aimed at both lower‐ and upper secondary school students. The much more common participation of those in upper secondary school was likely due to differences in national curricula. The upper secondary school curriculum includes a mandate for upper secondary schools to both have an active cooperation with universities and include experimental work in each biology course, whereas the lower secondary school curriculum does not. Thus, the project provided the participating teachers with a compact and easy way to fulfill those mandates. Specifically, rat tracks can be studied at any time of the year; thus, there were no time limitations on our part. In general, I found enthusiastic participation in the project by students in both lower‐ and upper secondary schools. Students often said that they liked participation as it was “something different than everyday school” (Aivelo and Huovelin 2020).

## 5 Conclusion

My study shows that the quality of the citizen‐collected data is not just a function of the skills (as through proxy of age) of the participating young people, but also of how they organize their tasks and how much help they receive from their teacher. Nevertheless, the young citizens provided reliable data on the occurrence and abundance of urban rats in their near environment. Observer quality remains a complex and poorly studied concept, especially concerning the effect of participants' attitudes. Nevertheless, this study provided a good starting point. While student's interest in the school subject and environmental issues correlated with the accuracy level, the attitude toward the focal species or disgust sensitivity did not seem to play a part in the quality of the data even though the studied species is commonly considered as an unloved species.

## Author Contributions

**Tuomas Aivelo:** conceptualization (equal), data curation (equal), formal analysis (equal), funding acquisition (equal), investigation (equal), methodology (equal), project administration (equal), resources (equal), visualization (equal), writing – original draft (equal), writing – review and editing (equal).

## Conflicts of Interest

The author declares no conflicts of interest.

## Supporting information


Appendix S1

## Data Availability

The data that support the findings of this study are openly available in Figshare at http://doi.org/10.6084/m9.figshare.19583206, whereas the code for the analysis is deposited in GitHub: https://github.com/aivelo/citsci.
