# Supplementary material for: How Do Age and Attitudes Affect the Quality of Data Collected by Young Citizen Scientists in an Ecological Research Project?
Source: Ecol Evol. 2024 Oct 10;14(10):e70428. doi: 10.1002/ece3.70428 (PMC11467035; doi:10.1002/ece3.70428)
Supplement: Supplementary file 1 — Appendix S1 [file ECE3-14-e70428-s001.pdf]

Supplementary data

How do age and attitudes affect the quality of data collected by young citizen scientists in an ecological research project?

Tuomas Aivelo, University of Leiden and University of Helsinki

Descriptive statistics of the data collection

Participation was focused on the first two terms (from August to November) and the last term of the school year (April and May). The school year is usually divided into five terms in most of the schools in Helsinki and generally the students participated within a month of the research lecture. The participating students were from Finnish, Swedish and English language schools but the questionnaire was only in Finnish; thus, a few students were excluded if they did not know Finnish. After data collection, each teacher described freely in an email whether the participation was voluntary or mandatory for students and whether they had additional incentives and how much help the students received in identifying and counting rat tracks. Both topics were clearly divided in three classes. In relation to the help, some student groups worked totally without any help from teacher during the identification and counting of rat tracks, while other could, if they wanted and needed, ask help from teacher. In contrast, some group identified and counted tracks all together in the classroom. The participation was mandatory in some student groups and totally voluntary in some groups. There were also groups that had voluntary participation, but students received extra credit for their biology course if they participated.

The questionnaire was administered post-research: at the beginning of the project in 2018, the Epicollect5 app had a direct link to the questionnaire which participants completed after sending data. Unfortunately, at the later stage from 2020 onwards, some of the operating systems did not work well with the link; thus, in later years, the link to the questionnaire was sent to the teachers after collection of the data. Direct link was used for first 58 % of the responses, whereas 42 % used the latter method. In some instances, students completed the questionnaire during classroom time, while some teachers asked students to complete this in their own time. As might be expected, this led to a lower participation rate, which could bias the results toward the more motivated students who completed the questionnaire.

The participating students were from Finnish, Swedish and English language schools but the questionnaire was only in Finnish; thus, a few students were excluded if they did not know Finnish. The demographics of the participating students was not specifically surveyed: the participating schools included both the most selective upper-secondary school and those lower-secondary schools that receive positive discrimination funding from the city due to having a substantial number of students from immigrant backgrounds.

Table S1: The number of samples per school type and the level of help students received and choice that they had on participation. Columns do not necessarily add up to the total as each teacher had consistent approach to help and choice during the study, but teacher could had different approaches within-schools and for some schools there were multiple teachers. Some teachers could teach in both lower and upper school. Total number of lower secondary schools in Helsinki is 73 and upper secondary schools 39.

School level	Variable		Number of schools	Number of teachers	Plates in total	Respondents in questionnaire
Lower	Help	No help	2	2	192	8
		If asked	2	2	60	0
		Done together	4	5	389	54
	Voluntarity	Mandatory	8	9	641	62
		Extra credit				
		Voluntary				
	Total		8	9	641	62
Upper	Help	No help	11	16	1438	260
		If asked	4	7	751	251
		Done together	2	3	176	72
	Voluntarity	Mandatory	12	21	1665	503
		Extra credit	2	2	692	80
		Voluntary	2	3	8	0
	Total		14	26	2365	583
Total			20	33	3006	645

Exploratory and confirmatory item response theory modelling

Table S2: Exploratory Factor Analysis results for all the initial items. Rotated factors loadings are shown for four factors: F1 matches to "Liking of biology", F2 to "Attitude towards rats" F3 to "Disgust", and F4 to "Attitude towards learning about pro environmental choices". Communality (h^2) is shown for all initial items. Items in italics are inversed.

Item	Statement	F1	F2	F3	F4	h^2
1	*I do not think that there is anything wrong with using rats in medical research.*	-0.04	-0.26	-0.14	-0.01	0.10
2	*I am not interested in learning how humans, plants and animals depend on each other.*	-0.27	-0.08	-0.05	-0.34	0.27
3	I like to study biology.	0.80	0.05	0.02	0.09	0.72
4	It would bother me tremendously to touch a dead body.	0.05	0.03	0.62	-0.06	0.40
5	It would bother me to see a rat run across my path in a park.	0.02	-0.29	0.35	0.08	0.18
6	I want to learn how energy can be saved and used in a more effective way.	0.02	-0.10	0.02	0.80	0.64
7	I think that rats belong in urban landscape.	0.00	0.35	-0.22	0.06	0.16
8	*It is boring to study biology.*	-0.73	-0.10	-0.03	-0.01	0.55
9	*Some aspects of biology can only be learned by dissecting preserved animals such as rats.*	0.01	-0.12	-0.17	0.04	0.05
10	I want to learn about climate change and how to prevent it.	0.03	0.13	0.14	0.66	0.55
11	I think it is perfectly acceptable for rats to be killed with traps or rodenticides in urban environments.	0.02	0.48	0.07	-0.01	0.24
12	I want to learn how new energy sources from sun, wind, tides, waves etc.	-0.02	0.02	-0.13	0.74	0.55
13	Biology is one of my favorite subjects in school.	-0.41	0.08	0.04	0.06	0.16
14	It would bother me to be in a science class, and to see a human hand preserved in a jar.	-0.03	0.01	0.54	0.04	0.30
15	I want to learn about technology helps us to handle waste, garbage and sewage.	-0.01	-0.05	-0.04	0.53	0.27
16	Usually we have interesting exercises in biology.	0.80	-0.08	-0.02	-0.06	0.61
17	*I might be willing to try eating rat meat under some circumstances.*	-0.06	0.05	-0.30	0.04	0.09
18	I would go out of my way to avoid walking through a cemetery.	0.02	0.00	0.26	0.02	0.07
19	For me biology is an easy school subject.	0.54	-0.09	-0.12	-0.06	0.31
20	*Basically, the city is a home for humans and we have the right to stop other animals from coming to our cities.*	-0.15	-0.10	-0.04	-0.09	0.06

Table S3: Confirmatory Factor Analysis results for all the initial items. Communality (h^2) and diagnostic values are shown for each items.

Item	Statement	h^2	RMSEAs x^2	p_{SCX^2}
1	*I do not think that there is anything wrong with using rats in medical research.*	0.13	0.03	0.37
2	*I am not interested in learning how humans, plants and animals depend on each other.*	0.20	0.03	0.07
3	I like to study biology.	0.67	0.03	0.37
4	It would bother me tremendously to touch a dead body.	0.51	0.03	0.07
5	It would bother me to see a rat run across my path in a park.	0.04	0.03	0.07
6	I want to learn how energy can be saved and used in a more effective way.	0.63	0.03	0.30
7	I think that rats belong in urban landscape.	0.03	0.00	0.64
8	*It is boring to study biology.*	0.49	0.03	0.37
10	I want to learn about climate change and how to prevent it.	0.44	0.03	0.11
11	I think it is perfectly acceptable for rats to be killed with traps or rodenticides in urban environments.	0.28	0.03	0.07
12	I want to learn how new energy sources from sun, wind, tides, waves etc.	0.51	0.03	0.07
13	Biology is one of my favorite subjects in school.	0.14	0.03	0.07
14	It would bother me to be in a science class, and to see a human hand preserved in a jar.	0.28	0.03	0.07
15	I want to learn about technology helps us to handle waste, garbage and sewage.	0.26	0.00	0.57
16	Usually we have interesting exercises in biology.	0.61	0.00	0.69
17	*I might be willing to try eating rat meat under some circumstances.*	0.06	0.03	0.19
18	I would go out of my way to avoid walking through a cemetery.	0.07	0.03	0.37
19	For me biology is an easy school subject.	0.30	0.03	0.07

The factors correlating with true and false positives and negatives

While the level of help, possibility of choice and age were significant variables for explaining the probability of true positives, true negatives or false negatives, none were significant for false positives (Table S4). The error rate in the plates that were linked to questionnaire responses was not statistically significant from the plates that could not be linked (i.e., questionnaire non-response) (mean 1.96 ± s.d. 7.32, vs. 1.78 ± 6.87, t_{3004}= 192.3, $p = 0.31$).

Table S4a: The variables affecting the occurrence of true positive track plate observation. The statistically significant factors were age which means that older participants were more likely to have true positive observations as did those who assessed plates together in the classroom. The baseline is female who did not receive extra help with mandatory participation.

Variable		Estimate	Std. Error	z value	p
**(Intercept)**		**-1.06**	**0.23**	**-4.62**	**<0.01**
**Age**		**0.37**	**0.13**	**2.87**	**<0.01**
**Help**	When needed	-0.37	0.27	-1.34	0.17
	**Done together**	**1.22**	**0.39**	**3.11**	**<0.01**
Choice	Voluntary with extra credit	-0.38	0.34	1.10	0.27
Gender	male	0.22	0.24	0.90	0.37
	not specified	1.05	0.58	1.81	0.07
	other	-0.26	3.37	0.02	0.98
Rat		0.21	0.16	1.32	0.19
Disgust		-0.11	0.14	-0.77	0.44
Biology		0.05	0.14	0.35	0.73
Environment		-0.05	0.15	-0.38	0.70

Table S4b: The variables affecting the occurrence of false positive track plate observation. No statistically significant factors found. The baseline is female who did not receive extra help with mandatory participation. When false positives were compared to only those reporting any tracks (true and false positives) or not having any tracks (false positives and true negatives), no significant variables were found.

Variable		Estimate	Std. Error	z value	p
**(Intercept)**		**-1.54**	**0.26**	**-5.77**	**<0.01**
Age		0.06	0.17	0.35	0.73
Help	When needed	0.01	0.31	0.05	0.96
	Done together	-0.34	0.52	-0.65	0.52
Choice	Voluntary with extra credit	-0.48	0.47	-1.02	0.31
Gender	male	0.21	0.28	0.75	0.42
	not specified	-0.20	15.3	-0.01	0.99
	other	-0.20	37.1	-0.01	0.99
Rat		0.04	0.19	0.23	0.82
Disgust		0.04	0.17	0.26	0.80
Biology		0.12	0.17	0.68	0.50
Environment		-0.20	0.18	-1.12	0.27

Table S4c: The variables affecting the occurrence of true negative track plate observation. The statistically significant factors were voluntary participation with rewards and assessing plates together in the classroom, which both led to smaller probability of true negative plates. The baseline is female who did not receive extra help with mandatory participation.

Variable		Estimate	Std. Error	z value	p
(Intercept)		0.09	0.20	0.45	0.65
Age		-0.19	0.12	-1.53	0.13
**Help**	When needed	0.32	0.24	1.34	0.18
	**Done together**	**-1.13**	**0.39**	**-2.87**	**<0.01**
**Choice**	**Voluntary with extra credit**	**-0.71**	**0.34**	**-2.05**	**0.04**
Gender	male	-0.24	0.22	-1.19	0.24
	not specified	-0.32	0.57	-0.55	0.57
	other	3.26	71.8	0.01	0.99
Rat		-0.09	0.15	-0.60	0.55
Disgust		0.06	0.13	0.49	0.62
Biology		-0.06	0.13	-0.46	0.65
Environment		0.08	0.14	0.58	0.59

Table S4d: The variables affecting the occurrence of false negative track plate observation. The only statistically significant factor was voluntary participation with rewards, which smaller probability of having false negative observation. The baseline is female who did not receive extra help with mandatory participation.

Variable		Estimate	Std. Error	z value	p
**(Intercept)**		**-3.90**	**0.76**	**-5.15**	**<0.01**
Age		-0.69	0.39	-1.76	0.08
Help	When needed	-0.27	1.10	-0.24	0.81
	Done together	0.39	1.06	0.36	0.71
**Choice**	**Voluntary with extra credit**	**-2.99**	**1.03**	**2.93**	**<0.01**
Gender	male	-0.58	0.59	-0.96	0.32
	not specified	-0.63	1.27	-0.49	0.62
	other	-2.01	72.4	-0.03	0.98
Rat		-0.62	0.40	-1.54	0.12
Disgust		-0.04	0.33	-0.12	0.91
Biology		-0.33	0.33	-1.02	0.31
Environment		0.59	0.36	1.64	0.10

The factors correlating with accuracy or success

When modelling accuracy by only those respondents whose plates did have confirmed rat tracks (n =163), any help or voluntary participation with rewards led to a more accurate reporting whereas males were less accurate than females (Table S5). In comparison, only rewards for voluntary participation and doing checking together correlated significantly with the likelihood of successful data collection as it lowered to success rate (Table S6). The effect of totally voluntary participation without any rewards could not be assessed in any different measures of accuracy as there was not enough cases and subsequently responses.

Table S5: The variables affecting the accuracy when only considering those track plates which had rat tracks based on the expert assessment. The statistically significant factors were the male gender which had substantially lower accuracy, voluntary participation with rewards and either having help when needed or assessing plates together in the classroom, which led to higher accuracy. The baseline is female who did not receive extra help with mandatory participation.

Variable		Estimate	Std. Error	z value	p
**(Intercept)**		**-1.04**	**0.35**	**-2.93**	**<0.01**
Age		-0.04	0.21	-0.20	0.84
**Help**	**When needed**	**-0.93**	**0.39**	**-2.36**	**0.02**
	**Done together**	**-1.62**	**0.71**	**-2.30**	**0.02**
**Choice**	**Voluntary with extra credit**	**-0.95**	**0.48**	**-1.99**	**0.05**
**Gender**	**male**	**1.01**	**0.36**	**2.78**	**<0.01**
	not specified	-0.55	1.18	-0.47	0.64
Rat		0.22	0.28	0.78	0.44
Disgust		0.30	0.24	1.23	0.22
Biology		0.30	0.19	1.54	0.12
Environment		-0.15	0.25	-0.58	0.56

Table S6: The variables affecting the success of the data collection. The significant variables were voluntary participation with rewards and when the track plate checking was done together, which both decreased success rate. The baseline is female who did not receive extra help with mandatory participation.

Variable		Estimate	Std. Error	z value	p
(Intercept)		0.71	0.34	2.06	<0.01
Age		0.09	0.18	0.49	0.75
**Help**	When needed	-0.28	0.64	-0.43	0.66
	**Done together**	**-0.22**	**0.64**	**-2.61**	**<0.01**
**Choice**	**Voluntary with extra credit**	**-1.25**	**0.47**	**-2.32**	**0.02**
Gender	male	-0.18	0.23	-0.80	0.43
	not specified	-0.39	0.58	-0.67	0.51
	other	-0.27	1.32	-0.67	0.84
Rat		-0.08	0.15	-0.51	0.61
Disgust		0.05	0.14	0.37	0.71
Biology		0.02	0.13	0.20	0.84
Environment		0.20	0.14	1.48	0.14

The effect of single or group work on the reliability

Anecdotally, the lower secondary school students worked more often in groups. To explore whether this affected the reliability of their results, I looked at the correlations and interactions between error rates, lower or upper school level and whether the students worked alone or in group. The data is not optimal for this analysis, as I do not have direct data on students group work. I assessed this indirectly so that any students who reported less than four plates, where inferred to work with other students, as the four plates was the standard number of plates on a site. It was more difficult to assess where the cases where all students working in the same group reported all four plates. I identified these cases in the data by looking at the reports of overlapping plate numbers in same time frames. This is prone to errors, though, as not all students answered to the questionnaire and thus some students, which I assessed as working alone where in fact working in group.

To assess the effects of school level and mode of working, I built generalized linear mixed models, which included the school as random factor and school level (lower/upper) and the mode of working (individual/group) as fixed factors with interaction. No variable was statistically significant: those participating alone had lower error rate (estimate: -0.92, standard error 0.55, $z = -1.6$, $p = 0.09$), the error rate was higher in upper secondary school (0.33, SE 0.39, $z = 0.8$, $p = 0.40$) and there was a positive interaction between working alone and upper secondary school (0.64, SE 0.633, $z = 1.0$, $p = 0.31$).

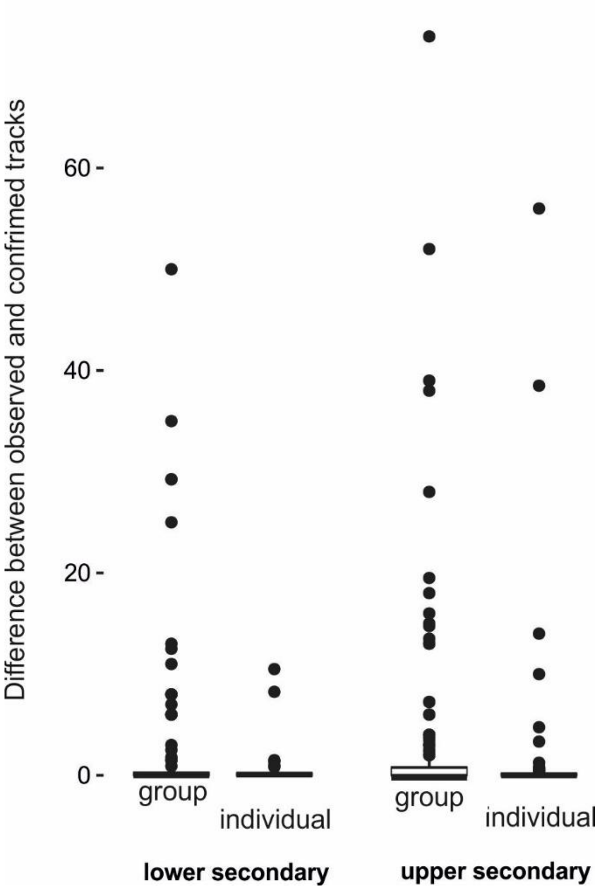

Figure S1: The error rate in lower and upper secondary group students depending whether they worked alone or as a group.

Translated questionnaire

This questionnaire relates to the rat track plate inquiry and it includes questions about your background, your relationship to nature and environment and school work.

This questionnaire will be used in Helsinki Urban Rat Project scientific research. We are studying how well a emotion-evoking urban animal suits as a target for inquiry in school science education. Further informatin can be obtained from .

The research consists of this questionnaire and no continuing participation is required.

We do not ask your name but rather the number of the plates you have used. These responses cannot be combined with personal data and they will not be published with the coordinates of your track plates. The responses will not be shared with teachers and participation is voluntary. You can decide not to participate without it having any effect on your school work.

I have read the information letter, acquired enough information on the research and consent that my answers can be used in scientific research.

Yes, I have not previously responded to this questionnaire.

Yes, I have previously responded to this questionnaire, with track plate codes

No.

The codes of the track plates that I have used

Age

Less than 14 years

14 years

15 years

16 years

17 years

18 years

19 years

More than 19 years

Gender

Male

Female

Other

Do not wish to tell

I do not think that there is anything wrong with using rats in medical research.

Totally disagree 1 2 3 4 5 Totally agree

I am not interested in learning how humans, plants and animals depend on each other.

Totally disagree 1 2 3 4 5 Totally agree

I like to study biology.

Totally disagree 1 2 3 4 5 Totally agree

It would bother me tremendously to touch a dead body.

Totally disagree 1 2 3 4 5 Totally agree

It would bother me to see a rat run across my path in a park.

Totally disagree 1 2 3 4 5 Totally agree

I want to learn how energy can be saved and used in a more effective way.

Totally disagree 1 2 3 4 5 Totally agree

I think that rats belong in urban landscape.

Totally disagree 1 2 3 4 5 Totally agree

It is boring to study biology.

Totally disagree 1 2 3 4 5 Totally agree

Some aspects of biology can only be learned by dissecting preserved animals such as rats.

Totally disagree 1 2 3 4 5 Totally agree

I want to learn about climate change and how to prevent it.

Totally disagree 1 2 3 4 5 Totally agree

I think it is perfectly acceptable for rats to be killed with traps or rodenticides in urban environments.

Totally disagree 1 2 3 4 5 Totally agree

I want to learn how new energy sources from sun, wind, tides, waves etc.

Totally disagree 1 2 3 4 5 Totally agree

Biology is one of my favorite subjects in school.

Totally disagree 1 2 3 4 5 Totally agree

It would bother me to be in a science class, and to see a human hand preserved in a jar.

Totally disagree 1 2 3 4 5 Totally agree

I want to learn about technology helps us to handle waste, garbage and sewage.

Totally disagree 1 2 3 4 5 Totally agree

Usually we have interesting exercises in biology.

Totally disagree 1 2 3 4 5 Totally agree

I might be willing to try eating rat meat under some circumstances.

Totally disagree 1 2 3 4 5 Totally agree

I would go out of my way to avoid walking through a cemetery.

Totally disagree 1 2 3 4 5 Totally agree

For me biology is an easy school subject.

Totally disagree 1 2 3 4 5 Totally agree

Basically, the city is a home for humans and we have the right to stop other animals from coming to our cities.

Totally disagree 1 2 3 4 5 Totally agree

Thank you for participating!
